# Fish gut microbiome and its application in aquaculture and biological conservation

**DOI:** 10.3389/fmicb.2024.1521048

**Published:** 2025-01-07

**Authors:** Nusrat Hasan Kanika, Nusrat Liaqat, Huifan Chen, Jing Ke, Guoqing Lu, Jun Wang, Chenghui Wang

**Affiliations:** ^1^Key Laboratory of Freshwater Aquatic Genetic Resources, Ministry of Agriculture and Rural Affairs, Shanghai Ocean University, Shanghai, China; ^2^National Demonstration Center for Experimental Fisheries Science Education, Shanghai Ocean University, Shanghai, China; ^3^Shanghai Engineering Research Center of Aquaculture, Shanghai Ocean University, Shanghai, China; ^4^Shanghai Collaborative Innovation for Aquatic Animal Genetics and Breeding, Shanghai Ocean University, Shanghai, China; ^5^National Experimental Teaching Demonstration Centre for Aquatic Sciences, Shanghai Ocean University, Shanghai, China; ^6^Department of Biology, University of Nebraska at Omaha, Omaha, NE, United States

**Keywords:** fish gut microbiome, aquaculture species, microbial diversity, host association, environment impact, conservation biology

## Abstract

Understanding the diversity and function of fish gut microbiomes has advanced substantially, yet many aspects remain poorly understood, particularly the interplay among microbiota, host species, and environmental factors in the context of conservation. This review explores the composition and abundance of gut bacterial communities in key aquaculture fish groups—cyprinids, ictalurids (catfish), salmonids, and cichlids (tilapia)—alongside the model organism zebrafish, across diverse geographic regions. The findings highlight environmental habitats and host species as primary determinants of gut microbiome structure, offering a global perspective on these microbial communities. Across all fish groups, the phyla Firmicutes, Fusobacteria, and Proteobacteria consistently dominated, while temperate, sub-equatorial, and sub-tropical regions exhibited the highest microbiome diversity, underscoring the contribution of taxonomic and environmental factors. The gut bacterial diversity of farm-raised fish shows a significant divergence from that of wild-caught fish, reflecting the impacts of ecological and management differences. Understanding the dynamic responses of fish gut microbiota is vital for guiding conservation efforts, safeguarding aquatic biodiversity, and advancing sustainable aquaculture practices. Future research should leverage innovative techniques and integrative approaches, both experimental and theoretical, to uncover the functional roles of microbiomes and predict their responses to environmental changes. Expanding geographic and taxonomic coverage will be critical for creating a comprehensive framework to inform global aquaculture and conservation strategies. Collectively, this perspective highlights the transformative potential of microbiome research in addressing global challenges in aquaculture and conservation biology.

## Introduction

Microbiomes play critical roles in maintaining ecosystem health, nutrient cycling, and climate regulation (Lennon et al., 2023). Within aquatic environments, fish, the most diverse group of vertebrates, are host to complex microbial communities that significantly impact their physiology and the health of the surrounding environment (Lorgen-Ritchie et al., 2023). The fish gut microbiome presents an invaluable window into host-microbiota-environment interactions, offering insights with direct implications for aquaculture and conservation. The composition of fish gut microbiota is shaped by a combination of environmental, biological, and behavioral factors, which collectively influence microbial communities across diverse fish species (Nayfach et al., 2021; Bertoncin et al., 2022). These microbiomes are sensitive to environmental conditions, including water temperature, oxygen levels, pH, and salinity, as well as intrinsic factors such as feeding behaviors, life stages, and various anthropogenic influences (Liu et al., 2016; Li et al., 2017a; Du et al., 2019; Zotta et al., 2019; Huang et al., 2020; Mukherjee et al., 2020). The dynamic interplay between fish and their gut microbiota plays a crucial role in shaping fish communities, making this an important area of study for better understanding and managing aquatic biodiversity.

Beyond contributing to essential ecosystem functions, such as nutrient cycling and climate regulation, gut microbiota are also critical for individual health. Alterations in microbial communities can affect phenotypic traits, immune mechanisms, and animal fitness in response to climate change, as physiological functions originate mostly from the gut (Dinan and Cryan, 2016; Mohajeri et al., 2018; Sepulveda and Moeller, 2020). Microbiomes are leveraged to enhance fatty acid production in muscle tissue and improve fish development (Eichmiller et al., 2016; Stephens et al., 2016; Mohajeri et al., 2018; Aldars-García et al., 2021; Asnicar et al., 2021; Chen et al., 2021; Zhang et al., 2022; Yin et al., 2023). The gut microbiome helps protect the intestinal barrier, prevent the overgrowth of opportunistic pathogens, and modulate the host immune system, all of which are crucial for maintaining fish health (Merrifield and Rodiles, 2015; Llewellyn et al., 2016; Nohesara et al., 2023). Conversely, disruption of the microbial balance can result in the proliferation of harmful bacteria, leading to disease outbreaks in aquaculture settings (Talwar et al., 2018; Vargas-albores et al., 2021; Wang et al., 2021). Therefore, understanding and manipulating the fish gut microbiome has become an important strategy for developing sustainable and disease-resistant aquaculture practices.

This review investigates how microbial abundance varies in response to temperature, habitat, and taxonomic differences across major fish groups in global aquaculture. We focus on cyprinids, ictalurids (catfish), salmonids, and cichlids (tilapia), which are economically significant aquaculture species (Lu and Luo, 2020). Additionally, we compare the gut microbiomes of farmed fish, which are raised in controlled environments with standardized diets, with those of wild-caught fish, which interact directly with their natural habitats, to understand how these contrasting conditions influence fish gut microbiomes. This comparative approach will help identify key environmental and dietary factors shaping the gut microbiome and highlight bacterial groups particularly sensitive to these variables. By highlighting these variations and their underlying causes, this review offers valuable insights into the role of the gut microbiome in promoting fish resilience and health under changing environmental conditions. These insights are essential for informing conservation strategies and optimizing sustainable aquaculture practices worldwide.

## Fish gut microbiomes varying across habitats, climatic zones, and feeding behaviors

**Figure 1:**
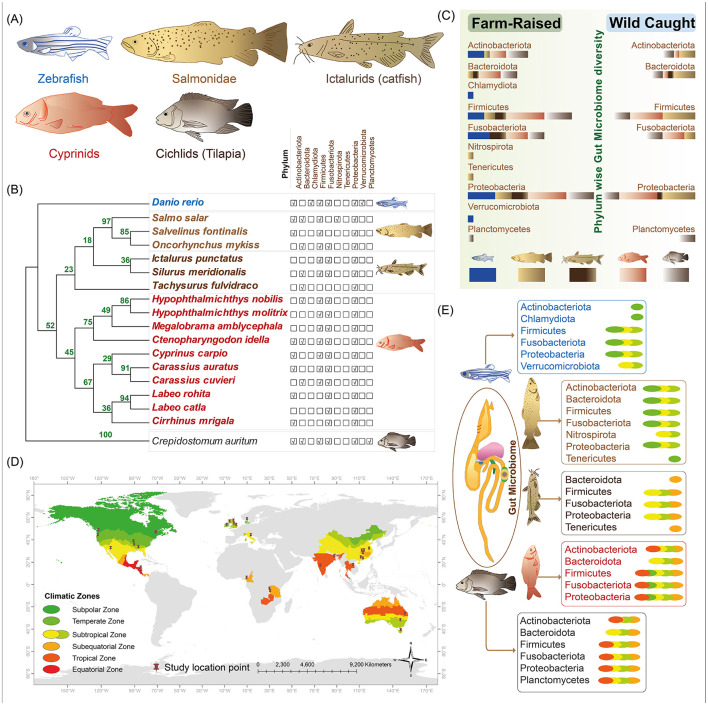
Gut microbiome diversity in four fish groups, farm-raised vs. wild-caught, and across climatic zones. **(A)** Fish groups and model species: four key fish groups commonly used in aquaculture—cyprinids, ictalurids (catfish), salmonids, and cichlids (tilapia)—are depicted, along with the model organism zebrafish. **(B)** Gut microbiome composition across different fish groups: phylogenetic relationships among the fish species are shown on the right, alongside dominant bacterial phyla associated with each fish species shown on the left. **(C)** Microbiome differences between farmed-raised and wild-caught fish: a bar chart illustrates the proportion of each bacterial phylum (represented by bar widths), with bar colors indicating specific fish groups. **(D)** Sampling sites and climate zones: sampling locations from reviewed studies are mapped globally, with climate zones differentiated by distinct colors. **(E)** Dominant bacterial phyla and climatic associations: dominant bacterial phyla in various fish groups are shown in relation to climate zones, with the same color scheme as **(D)**.

Gut microbiota composition varies among fish taxa, with hosts from the same taxonomic group generally exhibiting more similar gut microbiota than those from different groups; however, biological factors such as feeding habits can lead to remarkable differences within taxa (Huang et al., 2020). Distinct gut microbiome compositions were observed across different fish groups, with Proteobacteria, Fusobacteria, and Firmicutes being the most prevalent (Figures 1A, B). While Actinobacteria was present in cyprinids, salmonids, cichlids, and zebrafish, it was not reported in the catfish group (Figure 1B). Fish species exhibit distinct feeding behaviors across water layers, which shape their gut microbiome composition. Cyprinids, ictalurids (catfish), salmonids, cichlids, and zebrafish, ranging from bottom dwellers to surface feeders, display microbiota variations based on diet and ecological niches (Ang and Petrell, 1998; Rahman et al., 2008; Ramesh and Kiran, 2016; Thomas and Opeh, 2018). The variation in microbiota phyla highlights the impact of feeding behaviors on gut microbiome diversity (Figures 1B, C), emphasizing ecological adaptation (Sinha and Jones, 1967; Magoulick and Lewis, 2002; Watzin et al., 2008).

Studies also revealed the composition of fish gut microbiomes varies based on habitat characteristics and geomorphology, with factors like salinity and the differences between nearshore littoral and offshore profundal zones significantly influencing microbial diversity (Zotta et al., 2019; Huang et al., 2020; Sylvain et al., 2020; Kim et al., 2021; Shang et al., 2021). Farm-raised fishes in controlled environments show higher microbiome abundance, especially of Firmicutes, Fusobacteria, and Proteobacteria, compared to wild-caught fishes, where Firmicutes and Proteobacteria are most abundant (Figure 1C, Table 1).

**Table 1:** Microbial community composition and abundance in zebrafish, salmonidae, ictalurids (catfish), cichlids (tilapia), and cyprinids across various geographic and climate zones.

**Host**	**Habitat**	**Location**	**Climate zone**	**Dominant gut microbiota (phylum with *genus* or abundance)**	**References**
**Zebrafish** *Danio rerio*	Laboratory	Eugene, USA	Temperate	**Actinobacteriota***: Conexibacter, Mycobacterium, Pseudonocardia*, **Chlamydiota***: Neochlamydia, Parachlamydia*, **Fusobacteriota***: Cetobacterium*, **Proteobacteria***: Aeromonas, Catellibacterium, Comamonas, Delftia, Hyphomicrobium, Pelomonas, Pseudomonas, Raoultella, Shewanella, Sphingomonas, Stenotrophomonas, Thioprofundum, Vibrio, Yersinia*	Stagaman et al., 2017
		Quebec City, Canada	Temperate	**Actinobacteriota**: Abundant, **Firmicutes***:* Abundant, **Fusobacteriota**: Abundant, **Proteobacteria**: Mostly Abundant	Cornuault et al., 2022
		Eugene, OR, USA	Sub-tropical	**Firmicutes***: Streptococcus*, **Fusobacteriota**: *Cetobacterium*, **Proteobacteria**: *Aeromonas, Shewanella, Enterobacteriaceae, Diaphorobacter, Pseudomonas, Stenotrophomonas, Vibrio*	Stephens et al., 2016
		Shanghai, China	Sub-tropical	**Firmicutes***:* Abundant **Fusobacteriota***:* Mostly Abundant	Wang et al., 2021
		Birmingham (UAB), USA	Sub-tropical	**Actinobacteriota***: Bifidobacterium*, **Firmicutes***: Oscillospira, Ruminococcus, Anaeroglobus*, **Proteobacteria***: Pseudoxanthomonas, Legionella anisa, Legionella norrlandica*, **Verrucomicrobiota**: *Luteolibacter*	Koo et al., 2017
**Salmonids**					
Atlantic salmon (*Salmo salar*)	Wild caught	Jonesboro, USA	Temperate	**Firmicutes***: Peptostreptococcus, Streptococcus, Peptoniphilus, Gallicola, Peptococcus, Staphylococcus, Candidatus_Bacilloplasma, Bacillus, Shewanella* **Proteobacteria**: *Deefgea, Methylobacterium: Methylorubrum, Holosporaceae, Aeromonas*	Kara et al., 2021
Atlantic salmon (*Salmo salar*)	Farm-raised	Dover, Australia;	Temperate	**Bacteroidota***: Flavobacteriia, Cloacibacterium* **Firmicutes***: Clostridia, Vibrionaceae, Roseobacter, Bacillus, Aeribacillus, Anoxybacillus Geobacillus*, **Proteobacteria***: Vibrionaceae, Methylobacteriaceae*	Zarkasi et al., 2016
Atlantic salmon (*Salmo salar*)	Wild caught	Hirtshals, Denmark	Temperate	**Actinobacteriota***: Arthrobacter, Brevibacterium* **Firmicutes**: *Bacillus, Weissella, Leuconostoc, Lactobacillus, Pediococcus, Sporosarcina, Jeotgalicoccus, Streptococcus, Carnobacterium, Lactococcus, Ureibacillus, Geobacillus, Streptococcus* **Proteobacteria***: Erwinia, Sphingomonas, Pseudomonadales*	Gajardo et al., 2016
Atlantic salmon (*Salmo salar*)	Wild caught	Eastern Canada and Western Ireland	Temperate	**Actinobacteriota**: Abundant **Bacteroidota**: Abundant **Firmicutes**: *Mycoplasma*	Llewellyn et al., 2016
Atlantic salmon (*Salmo salar*)	Farm-raised	Aberdeen, UK	Temperate	**Bacteroidota**: Abundant **Firmicutes**: Abundant **Proteobacteria**: Abundant **Tenericutes**: Abundant	Dehler et al., 2017
Rainbow trout (*Oncorhynchus mykiss*)	Farm-raised	Büsum, Germany	Temperate	**Bacteroidota**: *Bacteroides, Porphyromonas*, **Firmicutes***: Staphylococcus, Vagococcus, Streptococcaceae, Lactobacillus, Lactococcus, Staphylococcus, Streptococcus* **Fusobacteriota***: Fusobacterium, Psychrilyobacter, Fusobacteriaceae*, **Proteobacteria***: Burkholderia, Aliivibrio fischeri, Acinetobacter johnsonii, Moritella, Photobacterium, Pseudoalteromonas, Shewanellaceae, Acinetobacter rhizosphaerae*	Ratten et al., 2017
Rainbow trout (*Oncorhynchus mykiss*)	Farm-raised	Argyll, UK	Temperate	**Firmicutes***: Lactobacillus, Acetanaerobacterium, Catellicoccus, Streptococcus, Weissella, Leuconostoc, Lactococcus, Enterococcus, Bacillus* **Proteobacteria***: Photobacterium, Pseudomonas, Acinetobacter, Maricurvus, Moritella, Pantoea*	Lyons et al., 2017
Atlantic salmon (*Salmo salar*)	Farm-raised	Washington, USA	Sub-tropical	**Actinobacteriota***: Actinomycetales* **Bacteroidota***: Sphingobacteriales, Flavobacteriales*, **Firmicutes**: *Lactobacillales*, **Nitrospirota**: *Nitrospirales*, **Proteobacteria***: Aeromonadales, Burkholderiales, Neisseriales, Aeromonas, Shewanella, Rickettsiales*	Schmidt et al., 2016
Brook trout (*Salvelinus fontinalis*)	Wild caught	North-western Italy, Italy	Sub-tropical	**Actinobacteriota**: Abundant **Firmicutes**: *Bacilli* **Fusobacteriota**: Abundant, **Proteobacteria**: Abundant	Mugetti et al., 2023
**Ictalurids (catfish)**					
Catfish (*Ictalurus punctatus*)	Farm-raised	Ferndale, USA	Sub-tropical	**Firmicutes***: Streptococcus, Lactobacillus* **Fusobacteriota***: Cetobacterium* **Proteobacteria***: Bradyrhizobium, Plesiomonas, Comamonadaceae, Enterobacteriaceae, Bradyrhizobium*	Bledsoe et al., 2016
Southern catfish (*Silurus meridionalis*)	Farm-raised	Wuhan, China	Sub-equatorial	**Bacteroidota**: Abundant **Fusobacteriota**: Abundant **Proteobacteria***: Enterobacteriaceae, Plesiomonas, unclassified Aeromonadaceae, Morganella* **Tenericutes**: Abundant	Zhang et al., 2018
Southern catfish, *Silurus meridionalis*	Farm-raised	Chongqing, China	Sub-equatorial	**Firmicutes***: Clostridiaceae, Clostridium, Bacillus* **Fusobacteriota***: Cetobacterium* **Proteobacteria***: Plesiomonas*	Zhang et al., 2017
Yellow catfish (*Pelteobagrus fulvidraco*)	Wild caught	Wuhan, China	Sub-equatorial	**Bacteroidota**: *Myroides* **Proteobacteria***: Plesiomonas, Yersinia, Enterobacter, Shewanella, Aeromonas, Vibrio*	Wu et al., 2010
**Cyprinids**					
Bigheaded carps (*Hypophthalmichthys* spp.)	Wild caught	Transdanubian, Hungary	Temperate	**Fusobacteriota***: Cetobacterium*, **Proteobacteria***: Pelomonas, Herbaspirillum, Aeromonas, Shewanella*	Borsodi et al., 2017
Crucian carp (*Carassius auratus*)	Wild caught	Yangtze River basin, China	Temperate	**Firmicutes***: Clostridium* XI, **Fusobacteriota***: Cetobacterium, Fusobacterium*, **Proteobacteria***: Aeromonas, Chitinibacter, Pseudomonas, Vibrio, Serratia*	Li et al., 2023
Bighead carp (*Hypophthalmichthys nobilis*), Silver carp (*Hypophthalmichthys molitrix*), Common carp (*Cyprinus carpio*), Goldfish (*Carassius auratus*), Freshwater drum (*Aplodinotus grunniens*)	Farm-raised and wild caught	Illinois River, USA	Temperate	**Firmicutes**: Abundant **Fusobacteriota**: Most Abundant^*^ **Proteobacteria**: Abundant	Eichmiller et al., 2016
Silver carp (*Hypophthalmichthys molitrix*)	Wild caught	Havana, IL, Louisiana, MO, West Lafayette, IN, and McBaine, MO, USA	Temperate	**Firmicutes***: Bacillus, Clostridium*, **Proteobacteria***: Aeromonas, Enterobacter*	Ye et al., 2014
Bighead carp (*Hypophthalmichthys nobilis*), Silver carp (*Hypophthalmichthys molitrix*), common carp (*Cyprinus carpio*)	Farm-raised and wild caught	Illinois River, USA	Sub-tropical	**Firmicutes**: Abundant, **Fusobacteriota**: Most Abundant,^*^ **Proteobacteria**: Abundant	Eichmiller et al., 2016
Crucian carp (*Carassius auratus*)	Farm-raised	Jiangsu, China	Sub-tropical	**Firmicutes***: Holdemania, Lactococcus, Staphylococcus*, **Fusobacteriota**: Cetobacterium, **Proteobacteria***: Vibrio, Aeromonas, Shewanella*	Li et al., 2017a
Herbivorous grass carp (*Ctenopharyngodon Idellus*) and *Carnivorous Siniperca chuatsi*, and *Silurus meridionalis*)	Wild caught	Wuhan, China	Sub-tropical	**Bacteroidota***: Bacteroides*, **Firmicutes***: Lactococcus, Clostridium, Proteocatella, Anaerorhabdus, Clostridium*, **Proteobacteria**: *Acinetobacte, Aeromonas, Serratia, Steroidobacter, Dechloromonas*	Yan et al., 2016
Cyprinid Fishes herbivorous grass carp (*Ctenopharyngodon idellus*) and blunt snout bream (*Megalobrama amblycephala*); omnivorous crucian carp (*Carassius auratus*); filter:feeding silver carp (*Hypophthalmichthys molitrix*) and bighead carp (*Hypophthalmichthys nobilis*)	Farm-raised	Wuhan, China	Sub-tropical	**Proteobacteria:** *Vibrio, Aeromonas, Shewanella*	Li et al., 2017b
Transgenic common carp (*Cyprinus carpio* L.)	Farm-raised	Guanqiao, China	Sub-tropical	**Bacteroidota**: Abundant **Firmicutes**: Abundant	Li et al., 2013
Grass carp (*Ctenopharyngodon idellus*)	Farm-raised	Jingzhou, Hubei, China	Sub-tropical	**Actinobacteriota***: Actinomyces* **Firmicutes***: Clostridium* **Proteobacteria***: Citrobacter*	Wu et al., 2012
Grass carp (*Ctenopharyngodon idellus*), Crucian carp (*Carassius cuvieri*), and Bighead carp (*Hypophthalmichthys nobilis*)	Farm-raised	Wuhan, China	Sub-tropical	**Bacteroidota***: Bacteroides*, **Firmicutes***: Clostridium, Proteocatella* **Fusobacteriota***: Cetobacterium*, **Proteobacteria***: Aeromonas*	Li et al., 2015
Gibel carp (*Carassius auratus gibelio*)	Farm-raised	Wuhan, China	Sub-tropical	**Actinobacteriota**: *Catellibacterium*, **Firmicutes**: *Cetobacterium, Holdemania, Lactococcus, Staphylococcus* **Proteobacteria**: *Pseudomonas, Acinetobacter, Serratia, Shewanella, Aeromonas, Roseomonas, Ensifer, Bose*	Li et al., 2017c
Grass carp	Farm-raised	Wuhan, China	Sub-equatorial	**Actinobacteriota***: Adlercreutzia*, **Bacteroidota***: Chryseobacterium, Citrobacter*, **Firmicutes***: Enterococcus*	Xiong et al., 2022
Grass carp (*Ctenopharyngodon Idellus*)	Wild caught	Wuhan, China	Sub-equatorial	**Firmicutes** ***:** Lactococcus, Leuconostoc, Weisella*	Yan et al., 2016
*M. amblycephala* and *C. idellus, S. chuatsi* and *C. alburnus*, omnivorous C. *carpio and C. auratus*	Wild caught	Wuhan, China	Sub-equatorial	**Firmicutes***: Clostridium* **Fusobacteriota**: *Leptotrichia, Cetobacterium* **Proteobacteria**: *Citrobacter*	Liu et al., 2016
Transgenic Common Carp (*Cyprinus carpio L*.)	Farm-raised	Guanqiao, China	Sub-equatorial	**Bacteroidota**: Abundant **Firmicutes**: Most Abundant^*^	Li et al., 2013
Indian major carps (IMCs), rohu (*Labeo rohita*), catla (*Catla catla*) and mrigal (*Cirrhinus mrigala*)	Farm-raised	West Bengal, India	Tropical	**Fusobacteriota**: *Fusobacterium*, **Proteobacteria**: *Aeromonas*	Mukherjee et al., 2020
Catla *(Cyprinus catla)*, Common carp (*Cyprinus carpio)*, mrigal *(Cyprinus mrigala)* and Rohu *(Labeo rohita)*	Wild caught	Maharashtra, India	Tropical	**Actinobacteriota**: Abundant **Firmicutes**: *Bacillus, Clostridium, Lactococcus* **Proteobacteria**: *Sphingomonas*	Pingle and Khandagle, 2023
**Cichlidae (Tilapia)**					
*Amphilophus* sp.	Wild caught	Nicaragua and Maganua, USA	Tropical	**Proteobacteria**: Most Abundant^*^ **Fusobacteria**: Most Abundant^*^ **Firmicutes**: Abundant **Bacteroidetes**: Abundant **Planctomycetes**: Abundant	Baldo et al., 2019
African cichlid	Wild caught	Tanganyika, Zambia; Barombi Mbo, Cameroon	Sub-equatorial	**Fusobacteria**: *Plecodus* sp.*, Xenotilapia* sp.*, Myaka* sp. **Proteobacteria**: *Lamprologus* sp.*, Variabilichromis* sp.*, Sarotherodon* sp., **Firmicutes**: *Lepidiolamprologus* sp.*, Neolamprologus* sp.*, Sarotherodon* sp. **Planctomycetes**: *Lepidiolamprologus* sp.*s, Interochromis* sp.*, Konia* sp. **Actinobacteria**: *Altolamprologus* sp.*, Ophthalmotilapia* sp.*, Sarotherodon* sp. **Verrucomicrobia**: *Enantiopus* sp.*, Eretmodus* sp. **Chlamydiae**: *Gnathochromis* sp.*, Simochromis* sp. **Bacteroidetes**: *Cyprichromis* sp.*, Konia* sp. **Chloroflexi**: *Neolamprologus* sp.*, Pungu* sp.	Baldo et al., 2017
	Wild caught	Tanganyika, Zambia and Tanzania	Sub-equatorial	**Fusobacteria**: *Cetobacterium* **Firmicutes**: *Clostridium, Turicibacter, Clostridium* XI, Lachnospiraceae (family), Clostridiales (order), Clostridiaceae (family), *Bacillus*, **Proteobacteria**: *Plesiomonas, Aeromonas*, Neisseriaceae (family), *Achromobacter*, **Planctomycetes**: Pirellulaceae (family)	Baldo et al., 2015
Nile tilapia (*Oreochromis niloticus*)	Farm-raised	Darmstadt, Germany	Sub-tropical	**Actinobacteriota**: *Arthrobacter, Chitinilyticum, Leucobacter, Luteitalea* **Proteobacteria**: Most Abundant^*^Acinetobacter, Aeromonas, Aquabacterium, Dechloromonas, Pseudomonas, Psychrobacter, Reyranella, Shewanella, Stenotrophomonas **Firmicutes**: *Lactobacillus, Lactococcus, Staphylococcus* **Bacteroidetes**: *Chryseobacterium, Dinghuibacter, Flavobacterium* **Planctomycetes**: *Blastopirellula* **Verrucomicrobia**: Abundant	Guimarães et al., 2021
Nile tilapia (*Oreochromis niloticus*)	Farm-raised	Gaozhou, China	Sub-tropical	**Firmicutes**: *Clostridium_sensu_stricto_2, Faecalibacterium, Hathewaya, f__Clostridiaceae_1_Unclassified, Terrisporobacter* **Proteobacteria**: *Escherichia–Shigella, Acinetobacter, Aeromonas* **Bacteroidota**: *f__Muribaculaceae_Unclassified, Bacteroides*	Kuebutornye et al., 2020
Nile tilapia (*Oreochromis niloticus*)	Farm-raised	Charoen, Thailand	Tropical	**Fusobacteria**: *Cetobacterium* **Proteobacteria**: *Aquaspirillum, Edwardsiella, Plesiomonas, Balneimonas, Rhodobacter* **Firmicutes**: *Weissella, Bacillus, Staphylococcus* **Actinobacteria**: *Corynebacterium*	Adeoye et al., 2016
Nile tilapia (*Oreochromis niloticus*)	Farm-raised	Sonora, Mexico	Sub-tropical	**Proteobacteria**: Most Abundant^*^ **Fusobacteria**: Most Abundant^*^ **Actinobacteria**: Abundant **Firmicutes**: Abundant	Martinez-Porchas et al., 2023
Nile tilapia, (*Oreochromis niloticus*)	Farm-raised	Plymouth University, UK	Temperate	**Actinobacteria**: *Mycobacterium, Propionibacterium, Curtobacterium, Phyciococcus, Corynebacterium* **Proteobacteria**: *Acinetobacter, Cobetia, Legionella, Plesiomonas, Janthinobacterium, Sphingomonas, Paracoccus, Methylobacterium, Rhodoplanes, Hyphomicrobium, Bradyrhizobium, Afipia* **Fusobacteria**: *Cetobacterium* **Firmicutes**: *Streptococcus, Weissella, Leuconostoc, Pediococcus, Lactobacillus, Enterococcus, Staphylococcus, Bacillus, Veillonella* **Bacteroidetes**: Other (Order Clostridiales), Other (Family Peptostreptococcaceae) **Spirochaetes**: SMB53 **Cyanobacteria**: *Streptophyta*	Standen et al., 2015

Temperature is another key factor influencing gut microbiome composition across climate zones. Fish from warmer environments often display greater microbial diversity, with temperature playing a crucial role in shaping species-specific responses (Wong and Rawls, 2012; Kokou et al., 2018). For example, yellow-tail kingfish showed higher gut microbiota richness at 26°C than at 20°C (Soriano et al., 2018), while turbot exhibited greater diversity at 20°C (Guerreiro et al., 2016). In rainbow trout, higher temperatures were associated with a reduction in Firmicutes (Huyben et al., 2018), and in salmon, higher temperatures led to a decrease in Acinetobacter and an increase in pathogenic *Vibrio* (Ley et al., 2008). Such variations underscore important role of temperature in influencing microbiota, particularly in temperature-sensitive fish (Chevalier et al., 2015). Among those climatic zones, fish from the subtropical region displayed the highest microbial diversity, with tropical, temperate, and sub-equatorial regions following diversity levels (Figures 1D, E, Table 1).

## Gut microbiome: an indicator for fish conservation and management strategies

The gut microbiome is increasingly recognized as an important indicator of environmental health and the adaptability of fish populations, offering valuable insights for conservation efforts (Soh et al., 2024). Factors such as geographic location, exposure to contaminants, urbanization, and the introduction of invasive species can significantly disrupt the gut microbiome, impacting essential processes like digestion, metabolism, immunity, and overall health (Zhu et al., 2021a; Clough et al., 2023; Lennon et al., 2023; Lorgen-Ritchie et al., 2023). Such disruptions complicate conservation strategies, particularly for endangered species, by altering microbial diversity and reducing adaptability. Shifts in microbial diversity in response to environmental pollutants or habitat changes can provide early warnings of ecological imbalance, facilitating timely conservation interventions (Zhu et al., 2021a).

Invasive species, such as Nile tilapia, illustrate how microbiome diversity can confer competitive advantages. Compared to native fish, invasive tilapia display higher gut microbial alpha diversity, reduced interspecies microbial competition, and enhanced food utilization, supporting niche expansion and local adaptation (Gu et al., 2020). Similarly, bighead carp and silver carp, major aquaculture species in East Asia, have become invasive in North America, where hybridization in the Mississippi River Basin (MRB) has further increased their adaptability (Wang et al., 2020). Studies suggest that hybrids benefit from a diversified gut microbiome, which, along with genomic adaptability, may facilitate invasion by supporting survival and local adaptation (Wang et al., 2020; Zhu et al., 2021b). These findings underscore the critical role of the gut microbiome in driving ecological success and adaptability in invasive species. As environmental pressures intensify, understanding the interactions between host genetics and microbiome diversity will be essential for managing invasive populations and protecting native biodiversity.

Managing gut microbiota in captive breeding programs can improve reintroduction success rates by enhancing the resilience and ecological fitness of released fish in their natural habitats (Zhu et al., 2021a). Microbiome-based interventions, including probiotics and prebiotics, hold considerable promise for enhancing resilience and adaptability in both farmed and wild fish, reducing mortality rates and reinforcing conservation outcomes (Jin Song et al., 2019; Vargas-albores et al., 2021; de Jonge et al., 2022). This approach is essential for species recovery, as a well-balanced gut microbiome strengthens adaptability, immune function, and overall health in reintroduced populations, enabling them to thrive in challenging environments.

Advancing sustainable aquaculture and implementing effective conservation strategies for vulnerable fish populations hinges on a comprehensive understanding of gut microbiome dynamics. Research has shown that gut microbiota plays a fundamental role in host resilience, immune function, and adaptation to environmental changes, all of which are critical for both farmed and wild fish (Fonseca and Fuentes, 2023; Zhu and Wang, 2023). Fish migration and breeding activities are closely associated with variations in gut microbiome composition, as they drive physiological and environmental changes (Llewellyn et al., 2016; Hamilton et al., 2019; Liu et al., 2021). As conservation and aquaculture practices increasingly incorporate microbiome management, this could become a powerful tool for sustaining biodiversity, aiding species recovery, and supporting sustainable aquaculture.

Embracing microbiome-based solutions strengthens the health and adaptability of individual species and contributes to the stability of entire aquatic ecosystems. Such microbiome-focused approaches have the potential to transform conservation practices by enhancing species survival, mitigating the impacts of invasive species, and restoring ecosystem health. By integrating these strategies, conservation efforts can foster resilient and balanced aquatic environments, paving the way for the long-term sustainability of our aquatic ecosystems.

## Discussion and future directions

This review provides a global perspective on the gut microbiome of four major aquaculture fish groups: cyprinids, ictalurids (catfish), cichlids (tilapia), and salmonids. It highlighted microbial composition and diversity across geographic regions and contrasting farmed and wild environmental conditions (Figure 1). By focusing on these important species, we identified dominant phyla and critical patterns that consistently appear across studies. Notably, samples from temperate, sub-equatorial, and subtropical zones exhibited the highest microbial diversity, emphasizing the interplay between taxonomic and environmental factors in shaping the microbiome (Figure 1E, Table 1). These findings underscore the adaptive significance of the gut microbiome in supporting essential functions such as digestion, immunity, and overall health, thereby enhancing the resilience and productivity of aquaculture species.

### Limitations and opportunities

While this review provides valuable insights, it is important to acknowledge its limitations. The selection of species and groups, while reasonable, does not fully represent the diversity of fish (Riera and Baldo, 2020). This limitation may restrict the generalizability of our findings, as different species and habitats harbor distinct microbiome compositions. Even within a single taxonomic group, various species inhabiting different niches can possess diverse gut microbiomes, which might have been underestimated in our review. Future research should aim to expand the analysis to include all species and taxonomic groups studied.

Additionally, the focus on microbial composition at the phylum level, while informative, represents a much higher taxonomic resolution. Comparisons at the genus or even at the species level would offer more detailed and biologically meaningful insights, particularly for understanding functional relationships within the microbiome. Furthermore, variability in sampling methods, sequencing depth, and environmental contexts across the reviewed studies introduces potential biases. This heterogeneity complicates cross-study comparisons and limits the ability to draw broad, definitive conclusions. A meta-analysis of raw sequencing data could address these inconsistencies, yielding more robust and statistically validated insights.

A geographic bias in sampling is evident, with most studies conducted in North America and East Asia, while regions such as Central Europe and Africa are underrepresented (Figure 1D, Table 1). This imbalance, similar to patterns observed in the Earth Microbiome Project (Gilbert et al., 2018), underscores the need for increased geographic diversity to achieve a truly global perspective on fish gut microbiomes. Expanding research efforts to include diverse regions and underrepresented species will be crucial for creating a comprehensive and unbiased framework for understanding and leveraging microbiome data in aquaculture and conservation.

### Addressing microbiome complexities with innovative, integrated approaches

The study of fish gut microbiomes is inherently complex due to the various influencing variables, such as diet, water quality, temperature, and salinity, which complicate efforts to isolate specific factors affecting microbial community structure (Egerton et al., 2018). The gut microbiome interacts dynamically with the host and the environment, requiring research to move beyond simple associations to uncover complex causal relationships (Xiong et al., 2019). The functional impact of the microbiome depends on the entire ecological network, where diet, environmental factors, and microbial composition interact in interdependent ways (Talwar et al., 2018; Diwan et al., 2022).

To address these challenges, the holobiont approach and multi-omics techniques should be employed. Future research on fish can follow the lead of large-scale microbiome initiatives, such as the Human Microbiome Project (Turnbaugh et al., 2007), to advance fish microbiome studies. For instance, the holobiont model has been used to explore how microbiomes interact with host genomes to drive adaptability and invasiveness in hybrid bighead and silver carp within the Mississippi River Basin (Wang et al., 2020; Zhu et al., 2021b). The integration of multi-omics data can uncover the functional roles of microbiomes in fish health and adaptation by linking microbial genes and metabolic pathways to host physiological traits, such as digestion efficiency, immunity, and stress tolerance.

Beyond experimental and field research, computational modeling and theoretical studies are crucial for enhancing our understanding of microbiomes as integral components of complex ecological networks (Kumar et al., 2019). Modeling microbial interaction networks, predicting the responses of these networks to environmental changes, and simulating diverse scenarios to assess the far-reaching impacts of the microbiome on host resilience and aquaculture productivity are promising avenues for future work. By combining experimental and computational approaches, researchers can unravel the intricate interdependencies among microbiomes, host, and environments, providing deeper insights into ecosystem functioning and advancing practical applications.

### Perspectives in aquaculture and conservation

Aquaculture practices, such as integrated pond fish farming and natural fish germplasm resource conservation, have been pivotal in sustainable aquaculture in regions like China (Li et al., 1990; Lu et al., 1997, 2020). Microbiome is likely to play a crucial role in these processes. Comparative analyses of gut microbial communities between natural and pond-cultured populations, as well as within the same pond ecosystem, could unveil the underlying mechanisms.

Further studies on the role of the microbiome in fish nutrition and health are necessary to enhance aquaculture productivity and promote healthier aquatic ecosystems. Developing microbiome-targeted feeds enriched with prebiotics, probiotics, or synbiotics can stimulate beneficial gut microbes and improve fish growth and disease resistance. For example, utilizing probiotics and incorporating alternative protein sources, such as insect meals, can enhance gut health and optimize aquaculture practices (Fonseca and Fuentes, 2023; Hasan et al., 2023). Feed formulations should be optimized based on microbiome profiles to improve nutrient uptake and resilience to pathogens. Insights from studies on wild and hybrid species can inform the design of functional feeds and strategies to enhance aquaculture productivity (Reshma et al., 2018; Cui et al., 2022).

Applying microbiome research to improve recirculating aquaculture systems by optimizing microbial communities in biofilters and water systems is crucial (Rurangwa and Verdegem, 2015; Mugwanya et al., 2021). Studying sediment and water column microbiomes to enhance nutrient recycling and minimize environmental impact is also essential. Promoting integrated multi-trophic aquaculture systems that leverage microbiome interactions across species can contribute to sustainable aquaculture practices (Troell et al., 2009).

Microbiome research is critical for understanding and maintaining wild fish populations. Future research should study the microbiomes of wild fish populations to understand their role in species health and ecosystem stability. Using microbiome monitoring to support species reintroduction programs and mitigate the impacts of invasive species is crucial. Enhancing biodiversity conservation by protecting critical microbial symbionts associated with endangered species is imperative.

## Conclusion

The fish gut microbiome represents a promising frontier for advancements in aquaculture and conservation biology. By harnessing the power of microbiome research, we can develop more sustainable aquaculture practices, enhance the resilience of wild fish populations, and safeguard the delicate balance of aquatic ecosystems. Interdisciplinary approaches and innovative technologies will pave the way for transformative solutions to global challenges in aquaculture sustainability and biodiversity conservation.
